# Type 17 immunity: novel insights into intestinal homeostasis and autoimmune pathogenesis driven by gut-primed T cells 

**DOI:** 10.1038/s41423-024-01218-x

**Published:** 2024-10-08

**Authors:** Daiya Ohara, Yusuke Takeuchi, Keiji Hirota

**Affiliations:** 1 Laboratory of Integrative Biological Science, Institute for Life and Medical Sciences, Kyoto University, Kyoto, Japan; 2 ImmunoSensation Cluster of Excellence, University of Bonn, Bonn, Germany

**Keywords:** IL-23, Th17, cDC_IL-23_, Inflammatory bowel diseases (IBD), Gut-joint axis, Gut-brain axis, Autoimmunity, Acute inflammation, Chronic inflammation

## Abstract

The IL-23 signaling pathway in both innate and adaptive immune cells is vital for orchestrating type 17 immunity, which is marked by the secretion of signature cytokines such as IL-17, IL-22, and GM-CSF. These proinflammatory mediators play indispensable roles in maintaining intestinal immune equilibrium and mucosal host defense; however, their involvement has also been implicated in the pathogenesis of chronic inflammatory disorders, such as inflammatory bowel diseases and autoimmunity. However, the implications of type 17 immunity across diverse inflammation models are complex. This review provides a comprehensive overview of the multifaceted roles of these cytokines in maintaining gut homeostasis and in perturbing gut barrier integrity, leading to acute and chronic inflammation in various models of gut infection and colitis. Additionally, this review focuses on type 17 immunity interconnecting multiple organs in autoimmune conditions, with a particular emphasis on the pathogenesis of autoimmune arthritis and neuroinflammation driven by T cells primed within the gut microenvironment.

## Introduction

Type 17 immunity is an immune response against certain types of pathogens that are not effectively controlled by classical type 1 or type 2 immunity. While type 1 immunity is involved primarily in combating intracellular pathogens, such as viruses and some bacteria, and type 2 immunity is required for defense against extracellular parasites, such as helminths, type 17 immunity is crucial for controlling extracellular bacteria and fungi that can cause mucosal tissue damage. Therefore, type 17 immunity is a specialized arm of the immune system that is mediated primarily by specific subsets of innate and T lymphocytes, including group 3 innate lymphoid cells (ILC3s) and IL-17-producing T helper 17 (Th17) cells. This type of immunity is characterized by the production of the signature cytokine IL-17A, along with other related cytokines such as IL-17F, IL-22, IL-23, and GM-CSF. These proinflammatory molecules mediate and sustain tissue inflammation by recruiting neutrophils and other immune cells to sites of infection or tissue injury.

Interleukin (IL)-23 is a heterodimeric cytokine composed of the proteins p19 and p40, which are encoded by *Il23a* and *Il12b*, respectively, and is essential for sustaining and amplifying type 17 immunity. While p19 is the unique subunit of IL-23, p40 is the common subunit shared with IL-12 [1]. A subset of mononuclear phagocytes (MNPs) is considered a key cellular source of IL-23 in response to various physiological and pathological stimuli. IL-23 signaling in both innate and adaptive immune cells is vital for mediating type 17 immunity, resulting in the upregulation of several type 17 cytokines [2]. These responses confer gut-protective effects but have also been implicated in the pathogenesis of inflammatory and autoimmune disorders. IL-23 promotes the activation of effector functions in ILC3s and Th17 cells, which both express high levels of the IL-23 receptor. Notably, downstream targets of IL-23, namely, IL-17A/F and IL-22, act on gut epithelial cells to promote wound healing and enhance gut barrier integrity by increasing the expression of mucus, antimicrobial peptides, chemokines, and tight junction proteins [3–5].

MNPs, which include monocytes, macrophages, and conventional dendritic cells (cDCs), play crucial roles in regulating the immune system under physiological and pathological conditions. In response to microbial infection, MNPs orchestrate innate and adaptive immune responses by sensing pathogens, upregulating costimulatory molecules and various inflammatory cytokines, including IL-23, mobilizing, and presenting foreign antigens to T cells, thereby driving the activation of innate immune cells and the differentiation of T helper (Th) cells [6].

In this review, we first describe the roles of type 17 immunity in governing host defense mechanisms and inflammatory conditions in the gut. We subsequently extended our scope to multiorgan networks, with a particular emphasis on T-cell-mediated autoimmune diseases.

### Type 17 immunity in maintaining intestinal homeostasis and controlling gut pathogens

#### IL-23 in gut homeostasis

Although the gastrointestinal tract harbors a myriad of commensal microorganisms, gut homeostasis is primarily sustained by basal immune responses to these microbes and the maintenance of physical barrier functions, effectively mitigating both local and systemic inflammation. IL-23 is constitutively detected in the gut but not in extraintestinal tissues such as the spleen, lung, liver, kidney, or skin [7]. Under physiological conditions, IL-23 is expressed primarily by cDCs within gut-associated lymphoid tissues such as mesenteric lymph nodes (mLNs), Peyer’s patches, and tertiary lymphoid organs, including isolated lymphoid follicles (ILFs) and cryptopatches (CPs), thereby maintaining gut homeostasis [8–10]. Activation of the intestinal IL-23‒IL-22 pathway regulates the abundance of specific commensal populations, such as segmented filamentous bacteria (SFB), capable of inducing nonpathogenic Th17 cells [11, 12].

#### IL-23‒IL-22 axis in the host defense against mucosal infections

##### The role of IL-22 and IL-17A/F in gut infection

The IL-23‒IL-22 axis is essential for the elimination of gut invasive pathogens, such as attached and effiving bacteria, by orchestrating the recruitment of neutrophils and inflammatory monocytes via the induction of chemokines, including CXCL1, CXCL2, CCL1, and CCL2, which are secreted by intestinal epithelial cells (IECs) in response to IL-22 stimulation. In addition, IL-22 signaling enhances the expression of RegIII, an antimicrobial peptide, and fucosyltransferase 2 (Fut2), which promotes the fucosylation of the luminal side of the IEC membrane [3, 13–15]. *Il23a*^−/−^ and *Il22*^−/−^ mice succumb to *Citrobacter. rodentium (C. rodentium)* infection, a model organism for human enteropathogenic *E. coli* infection [13, 16, 17]. The signaling pathway downstream of the IL-22 receptor in IECs has been well characterized [18]. The binding of IL-22 to its receptor induces the phosphorylation of JAK1 and TYK2 and subsequently phosphorylates STAT3. The dimerized pSTAT3 then translocates to the nucleus, where it induces the expression of chemokines and antimicrobial peptides. Additionally, IL-22 signaling activates the PI3K and MAPK pathways, which synergistically promote the expression of these genes together with pSTAT3.

IECs can be subdivided into two major branches: absorptive cells and secretory cells, the latter comprising goblet cells, Paneth cells, enteroendocrine cells, and tuft cells. Recent studies have highlighted the role of IL-22 signaling in different IEC subtypes. One report suggested that IL-22 signaling in absorptive cells is involved in the elimination of *C. rodentium* infections [19], whereas another study showed that IL-22 signaling in secretory cells, especially in goblet cells, is important for maintaining gut barrier integrity [20]. IL-22 signaling has also been implicated in the differentiation and maturation of Paneth cells, as shown by studies using Il22^−/−^ mice and human gut organoid models [15, 21]. However, further study is needed to elucidate the precise mechanisms by which IL-22 signaling regulates the specific functions of each IEC subset during the clearance of intestinal infections.

While IL-23 is known for its ability to stimulate IL-17A/F production from Th17 cells alongside IL-22, the efficacy of IL-17A/F in clearing *C. rodentium* is relatively modest in comparison to that of IL-22. Mice deficient in IL-17A and/or IL-17F signaling exhibit an increased bacterial burden during *C. rodentium* infection; however, all infected mice survive and maintain a normal body weight [13, 22]. Interestingly, the upregulation of IL-17A during *C. rodentium* infection occurs independently of IL-23 [13, 16], indicating the functional capacity of Th17 cell-derived IL-17A production machinery in the absence of IL-23, potentially under the regulation of alternative Th17-associated cytokines such as IL-6, TGF-β, and IL-1β. Conversely, the induction of IL-22 production by both ILC3s and Th17 cells is completely dependent on IL-23 [13]. Mechanistically, IL-22 induces the production of antimicrobial peptides, including RegIII and S100A proteins, from the intestinal epithelium, whereas IL-17A/F induces β-defensin production, which appears to be insufficient for controlling *C. rodentium* infection [22]. Thus, the IL-23‒IL-22 axis plays a more critical role in combating *C. rodentium* compared to the IL-23‒IL-17A/Faxis, which has distinct functions in maintaining gut homeostasis.

##### IL-23-producing cells in *C. rodentium* infection

The susceptibility of *Il23a*^−/−^ mice to *C. rodentium* infection has sparked considerable interest in identifying the specific intestinal cell subsets responsible for IL-23 production [16]. While IL-23 from bone marrow-derived CD11c^+^ MNPs, comprising cDCs and macrophages, is crucial for *C. rodentium* clearance in a CD11c-diphteria toxin receptor (DTR) mouse model [23], the dominant IL-23-producing subset within MNPs remains elusive because of the heterogeneity of cDCs and macrophages.

Gut cDCs can be subdivided into four subpopulations on the basis of the surface markers CD103 and CD11b. While CD103^+^ CD11b^-^ cDCs are classified as cDC1s, CD103^+^ CD11b^+^, CD103^-^ CD11b^+^, and CD103^-^ CD11b^-^ cDCs are classified as cDC2s [6]. The development of cDC1s and cDC2s relies on the transcription factors Batf3 and IRF4, respectively [24–26]. Ly6C^hi^ monocytes differentiate into Ly6C^-^ MHCII^+^ macrophages through the coexpression of Ly6C and MHCII [27, 28]. Although both cDCs and macrophages express high levels of CD11c and MHCII, they can be segregated using cDC-specific markers such as Zbtb46 and CD26 and macrophage-specific markers such as CD64 and CD88 [6, 29–32].

It was initially proposed that CD103^+^ CD11b^+^ cDC2s are the key source of IL-23 in the gut [33, 34]. CD11c^Cre^ *Notch2*^flox/flox^ mice lacking CD103^+^ CD11b^+^ cDC2s were shown to be highly susceptible to *C. rodentium* infection. Notably, mixed bone marrow chimeric mice generated from CD11c^Cre^ *Notch2*^flox/flox^ and *Il23a*^−/−^ bone marrow (BM) cells succumbed to *C. rodentium* infection, similar to those receiving only *Il23a*^−/−^ BM cells. These findings collectively underscore the indispensable role of IL-23 derived from Notch2-dependent CD11c^+^ MNPs, primarily CD103^+^ CD11b^+^ cDC2s, in conferring protection against *C. rodentium* infection. Conversely, another study investigated the role of CD103^+^ CD11b^+^ cDC2s as the primary source of IL-23 in the gut [35]. This study utilized bacterial artificial chromosome transgenic mice, called huLangerin-diphteria toxin (DTA) mice, which also lack gut CD103^+^ CD11b^+^ cDC2s similar to those of CD11c^Cre^ *Notch2*^flox/flox^ mice. Intriguingly, despite the depletion of gut CD103^+^ CD11b^+^ cDC2s in these mice, huLangerin-DTA mice were resistant to *C. rodentium* infection compared with WT mice, suggesting that alternative cellular sources capable of producing IL-23 distinct from CD103^+^ CD11b^+^ cDC2s.

**Fig. 1:**
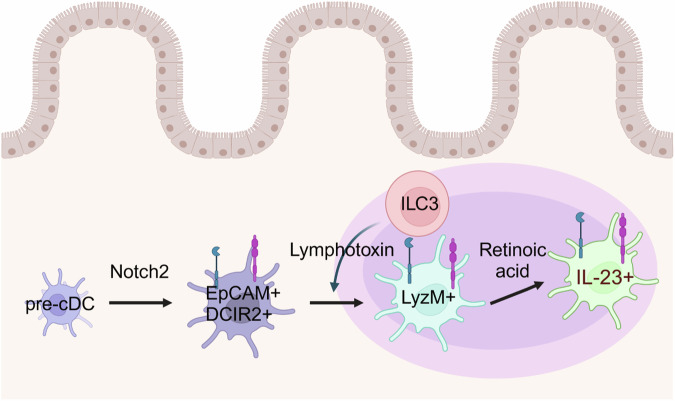
Three-step developmental model of IL-23-producing cDCs. The activation of Notch2 signaling serves as the initial trigger for the differentiation process of EpCAM^+^ DCIR^+^ cDCs. This is followed by the induction of CIA-DC signature genes, such as LyzM, in cDCs through lymphotoxins from ILC3s and their recruitment into tertiary lymphoid organs. Ultimately, the terminal differentiation of these cells necessitates the involvement of retinoic acid signaling, which is pivotal for endowing them with robust ability to produce IL-23

To further delineate the cellular source of IL-23, we generated *Il23a*^Venus^ mice, which allow direct visualization of IL-23-producing cells [8]. This strain showed that the majority of IL-23-producing cells were EpCAM^+^ DCIR2^+^ cDC2s, which can be divided into two subpopulations: CD103^+^ CD11b^+^ and CD103^-^ CD11b^-^. Notably, EpCAM^+^ DCIR2^+^ CD103^-^ CD11b^-^ cDC2s, termed cDC_IL-23_, exhibited greater potency in terms of IL-23 production than did the CD103^+^ CD11b^+^ cDC2 fraction during *C. rodentium* infection. Furthermore, we demonstrated that the development of the EpCAM^+^ DCIR2^+^ cDC2 subset depends on Notch2 signaling (Fig. 1). CD11c^Cre^ *Notch2*^flox/flox^ mice indeed lack IL-23 expression not only in CD103^+^ CD11b^+^ cDC2s but also in EpCAM^+^ DCIR2^+^ CD103^−^ CD11b^−^ cDC2s (cDC_IL-23_). IL-23 expression in cDC_IL-23_ is likely intact in Langerin-DTA mice [35]. The absence of the cDC_IL-23_ subset likely accounts for the high susceptibility of CD11c^Cre^ *Notch2*^flox/flox^ mice to *C. rodentium* infection [8, 34]. Consequently, our findings concerning cDC_IL-23_ provide insights that reconcile the discrepancy observed in the phenotypes of CD11c^Cre^ *Notch2*^flox/flox^ and huLangerin-DTA mice during *C. rodentium* infection.

**Fig. 2:**
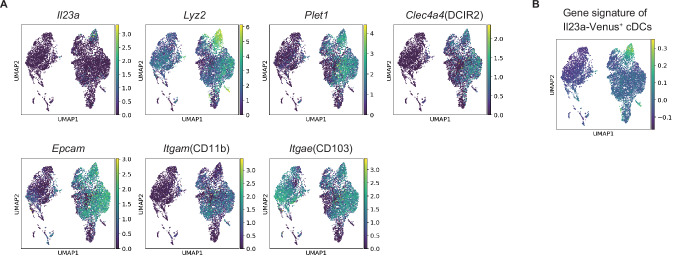
Similar gene expression profiles between Il23a-Venus^+^ EpCAM^+^ DCIR2^+^ CD103^-^ CD11b^-^ cDCs (cDC_IL-23_) and LyzM^hi^ CIA-DCs. **A** Reanalysis of the scRNA-seq atlas of SILP cDCs (E-MTAB-9522) [10]. Marker gene expression associated with CIA-DC and Il23a-Venus^+^ cDCs was projected via UMAP analysis of SILP cDCs. **B** The gene set score of the Il23a-Venus^+^ cDC signature was calculated via the scanpy.tl.score_genes function and projected via UMAP analysis [190]. The top 100 upregulated genes sorted by adjusted *p* value in bulk RNA-seq data of Il23a-Venus^+^ cDCs compared with CD103^+^ CD11b^+^ cDCs were used as the signature genes of Il23a-Venus^+^ cDCs (DRA016070) [8]

IL-23-producing cDCs are specifically localized in mLNs and tertiary lymphoid organs, such as ILFs and CPs, which harbor abundant numbers of IL-22-producing ILC3s [8]. Recently, a distinct cDC subpopulation called “CIA-DCs” was shown to be confined to ILFs and CPs [10]. These cDCs are defined as LyzM^hi^ Plet1^+^ CD103^-^ cDCs. Single-cell RNA-seq analysis of SILP cDCs revealed that LyzM^hi^ cDCs highly express *Il23a*, *Epcam*, *Clec4a4* (DCIR2), and other gene signatures akin to Il23a-Venus^+^ cDCs, indicating that LyzM^hi^ CIA-DCs likely include the majority of the cDC_IL-23_ population [10] (Fig. 2A, B). In addition, ILC3-derived lymphotoxin is essential for the development of LyzM^hi^ Plet1^+^ CD103^−^ cDCs, as CD11c^Cre^ *Ltbr*^*f*lox/flox^ mice lack these cDCs alongside some CD103^+^ CD11b^+^ cDC2s, resulting in decreased *Il23a* expression in the colon and increased bacterial burdens in the feces, liver, and spleen during *C. rodentium* infection compared with those in WT mice [36]. Taken together, these findings underscore the importance of cDC_IL-23_, which is located within gut-associated lymphoid tissues, in orchestrating mucosal host defense against *C. rodentium*.

Additionally, CX3CR1^+^ macrophages were proposed as a potential cellular source of IL-23 during *C. rodentium* infection via CX3CR1-DTR generated by crossing CX3CR1-flox-STOP-flox-DTR with CD11c^Cre^ or Rosa26-flox-STOP-flox-DTR mice with CX3CR1^Cre^ [23, 37]. The administration of diphtheria toxin to these mice enables the selective depletion of CX3CR1^+^ macrophages while preserving CD103^+^ CD11b^+^ cDC2s, increasing the susceptibility of the mice to *C. rodentium* infection. Furthermore, mixed bone marrow chimeric mice generated from CX3CR1-DTR and *Il23a*^−/−^ BM cells also succumbed to *C. rodentium* infection. However, CX3CR1 is also expressed by CD103^-^ cDC2s, suggesting potential depletion of IL-23-producing cDC2s in CX3CR1-DTR mice [38, 39]. Indeed, CX3CR1-Cre is expressed in CD103^-^ cDC2s from CX3CR1^Cre^ mice [23]. Therefore, it is necessary to consider that not only CX3CR1^+^ macrophages but also cDC_IL-23_ could be affected in CX3CR1-DTR mice. Given the absence of IL-23 reporter expression in gut macrophages [8], it is reasonable to assert that cDC_IL-23_, rather than CX3CR1^+^ macrophages, represents a key cellular subset crucial for IL-23 production in *C. rodentium* infection.

##### Spatiotemporal regulation of the IL-23‒IL-22 axis in *C. rodentium* infection

**Fig. 3:**
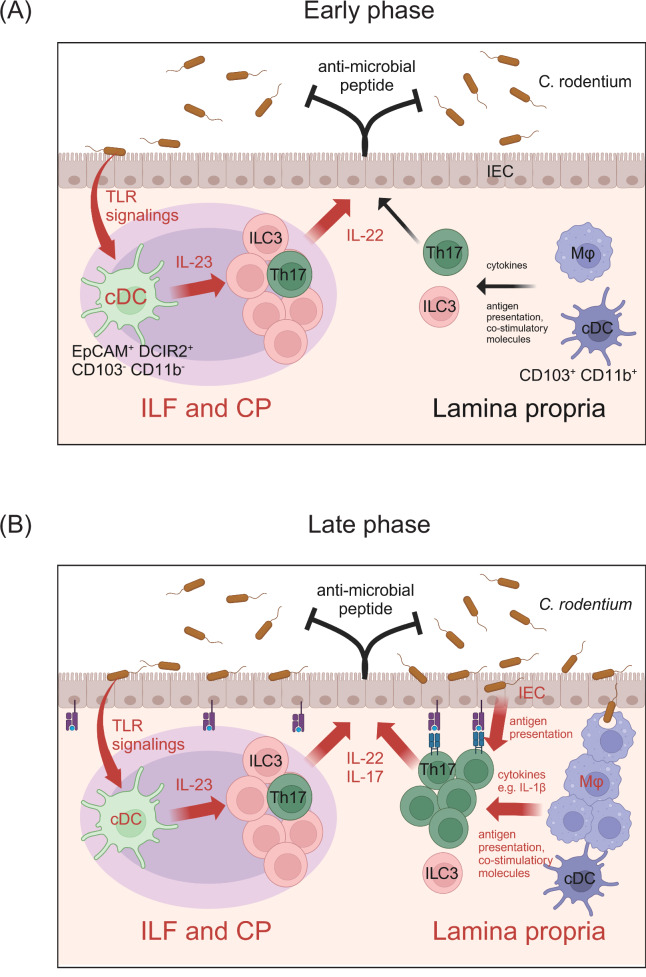
Spatiotemporal regulation of the IL-23‒IL-22 axis during *C. rodentium* infection. **A** During the early phases of *C. rodentium* infection, the IL-23‒IL-22 axis in tertiary lymphoid organs, such as ILFs and CPs, is critical. Specifically, IL-23-producing EpCAM^+^ DCIR2^+^ CD103^-^ CD11b^-^ cDCs (cDC_IL-23_) activated by TLR signaling via pathogen-associated molecular patterns play a pivotal role in stimulating IL-22 expression by ILC3s. **B** During its later phase, in addition to the interaction between cDC_IL-23_ and ILC3s in tertiary lymphoid organs, the engagement of macrophages and IECs in the activation of Th17 and/or ILC3s within the lamina propria emerges as an additional significant aspect in combating *C. rodentium* infection. Notably, CX3CR1^+^ macrophages and IECs facilitate IL-22 expression by Th17 cells through the presentation of antigens, costimulatory molecules, and cytokines such as IL-1β, thereby fostering sustained activation of gut epithelial cells and eventual elimination of *C. rodentium* infection

There seems to be complex regulation of the cellular origins of IL-23 and IL-22 during the early and late phases of *C. rodentium* infection. As described earlier, IL-23-producing cDCs in tertiary lymphoid organs detect *C. rodentium* and produce high amounts of IL-23 during the initial phase of infection. Subsequently, ILC3s in close proximity to such cDCs upregulate IL-22 expression in response to IL-23 (Fig. 3A). This cascade triggers the activation of IECs, instigating early host defense mechanisms characterized by the production of antimicrobial peptides and mucus, alongside the recruitment of inflammatory monocytes and neutrophils via chemokine signaling [40]. Notably, ~60% of *Plzf*^Cre^ *Il22*^flox/flox^ mice, which lack *Il22* in innate-type lymphoid cells such as ILCs, NKs, and γδTs but not in CD4^+^ T cells, died during the early stages of *C. rodentium* infection. Like *Il23a*^−/−^ and *Plzf*^Cre^ *Il22*^flox/flox^ mice [34, 41], mice with deficiencies in IL-23 production from cDCs, such as Zbtb46-DTR or CD11c^Cre^ *Notch2*^flox/flox^ mice, also succumb during the early phase of *C. rodentium* infection [34].

However, at later stages of infection, CD4^+^ T cells in the crypts emerge as the major sources of IL-22, as demonstrated by studies using CD4^Cre^ *Il22*^flox/flox^ mice [40], which specifically lack *Il22* in T cells. The absence of IL-22 in CD4^+^ T cells results in an inability to restrain the proliferation of *C. rodentium* during the late phase of infection, leading to ~40% mortality. In these mice, *C. rodentium* invasion extends into deep crypts as the infection progresses. Histologically, IL-23-producing cDCs are mainly localized within tertiary lymphoid structures, colocalizing with abundant ILC3s [8, 40], whereas macrophages are predominantly situated within the crypts [42]. These macrophages can regulate the effector functions of IL-22-producing CD4^+^ T cells during the late phase of infection. Intriguingly, compared with *Il23a*^−/−^ and Zbtb46-DTR mice, mice lacking macrophages, such as CX3CR1-DTR or MM-DTR mice, tend to succumb during the later phase of infection [23, 37, 41], underscoring the essential roles of both Th17 cells and CX3CR1^+^ macrophages in controlling *C. rodentium* infection during the late phase. It is tempting to speculate that differentiated Th17 cells in secondary lymphoid organs could interact with CX3CR1^+^ macrophages within crypts during the late phase of infection.

Additionally, it has been reported that IECs in crypts play a role in pathogen-derived antigen presentation via MHCII molecules, which in turn induces IL-22 production from CD4^+^ T cells [19]. *C. rodentium* is known to infect and attach to Ly6G^+^ distal colonocytes but not to FABP2^+^ proximal colonocytes, and its infection specifically upregulates MHCII expression predominantly in Ly6G^+^ distal colonocytes. A study using Villin^Cre^ MHCII^flox/flox^ mice revealed increased susceptibility to *C. rodentium* during the late phase of infection, phenocopying CD4^Cre^ *Il22*^flox/flox^ mice [40]. Notably, CD4^+^ T cells from *C. rodetium*-infected Villin^Cre^ MHCII^flox/flox^ mice exhibited reduced production of IL-22 and IL-17A during the late phase of infection.

These findings clearly demonstrate that, in addition to MNPs, IECs also promote microbicidal Th17 functions during *C. rodentium* infection. Taken together, the interaction of Th17 cells with both MNPs and IECs could potently lead to IL-22 production through direct antigen presentation, costimulation stimuli, and cytokine communication, ultimately contributing to the elimination of *C. rodentium* (Fig. 3B).

### Type 17 immunity in inflammatory bowel diseases

Inflammatory bowel disease (IBD) consists of Crohn’s disease (CD) and ulcerative colitis (UC), characterized by chronic and relapsing inflammation impacting the gastrointestinal tract, which affects millions of individuals worldwide. IL-23 plays a pivotal role in maintaining gut homeostasis and orchestrating immune responses against pathogens, such as attaching and effacing bacteria. However, excessive production of IL-23, triggered by genetic or environmental factors, can precipitate the onset and progression of IBD [43]. Consequently, antibody therapies targeting the IL-23 subunits p19 or p40 have shown remarkable efficacy in ameliorating IBD [44]. Understanding the sources of IL-23 production and its regulatory mechanisms and how IL-23 regulates type 17 immunity in chronic inflammation, particularly in comparison with acute inflammation during gut infections, is crucial for comprehensively delineating IBD pathology and advancing therapeutic interventions.

#### Roles of type 17 immunity in IBD pathogenesis

**Fig. 4:**
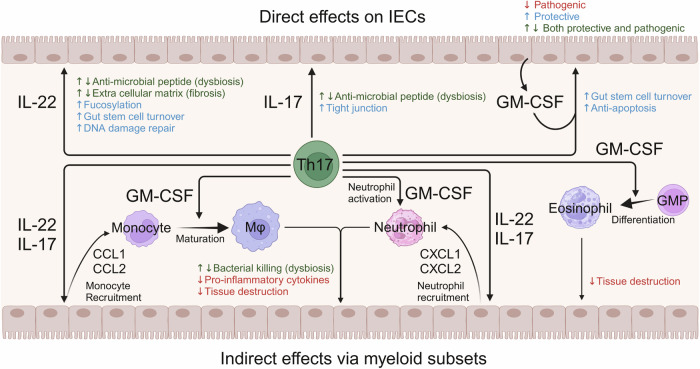
The opposing roles of Th17-producing cytokines in IBD pathogenesis. Th17-producing cytokines, including IL-22, IL-17, and GM-CSF, influence diverse biological processes and can act both protectively and pathogenically in inflammatory bowel disease (IBD). The effects of these cytokines can be categorized into direct effects on IECs and indirect effects via myeloid subsets. In the context of their direct effects on IECs, these cytokines regulate the gut microbiota through the production of antimicrobial peptides and increase the fucosylation of membrane proteins on IECs. These cytokines also promote gut stem cell turnover, inhibit apoptosis induced by injury, and strengthen tight junctions between IECs, thereby maintaining gut integrity. Collectively, these effects play a protective role in IBD. In the indirect effect mediated through myeloid cell subsets, IL-22 and IL-17 enhance chemokine production by IECs, which in turn recruit neutrophils and monocytes. GM-CSF is involved in neutrophil activation, monocyte maturation, and eosinophil differentiation. The recruited and activated myeloid cells contribute to bacterial killing through phagocytosis. However, these cells can also mediate inflammation via cytokine production (i.e., IL-23 and IL-1β) and tissue destruction, thereby exacerbating the pathology of IBD

The implications of type 17 immunity in the pathogenesis of IBD are complex and characterized by conflicting findings. To elucidate this complexity, we have summarized experimental data that evaluated the roles of IL-22, IL-17, and GM-CSF in three tables (Tables 1–3). We have also described the roles of these Th17-producing cytokines in the pathogenesis of human IBD. Furthermore, we explored the contribution of IFN-γ-producing exTh17 cells or Th1 cells induced by IL-23 to the pathogenic sequence of IBD. This section initially delineates the dual-edge roles of Th17-producing cytokines induced by IL-23 in IBD (Fig. 4), followed by an overview of recent insights into the mechanistic implications of IL-23 in the pathogenesis of IBD.

**Table 1:** The roles of IL-22 in colitis models

Article	Colitis model	Method	The role of IL-22	The role of IL-23	Ref
Sugimoto et al.	DSS	anti-IL-22 Ab	Protective		[45]
Moniruzzaman et al.	DSS	*Il22ra*^−/−^	Protective		[46]
Bishop et al.	DSS	anti-IL-22 Ab	Protective		[47]
Zenewicz et al.	DSS	*Il22*^−/−^	Protective		[48]
	naïve T Transfer	*Il22*^−/−^ T cells into *Il22*^−/−^ *Rag2*^−/−^	Protective		
	naïve T Transfer	*Il22*^−/−^ T cells into *Rag2*^−/−^	Not significant		
Gunasekera et all	*Il10* KO	*Il22*^−/−^	Inflammatory		[49]
Bernshtein et al.	*Cx3cr1*^Cre^ *Il10ra*^flox/flox^	*Il22*^−/−^	Inflammatory	Inflammatory	[50]
Morrison et al.	Hh + anti-IL-10	anti-IL-22 Ab	Inflammatory		[51]
Wang et al.	Hh infection in 129*Rag2*^−/−^	anti-IL-22 Ab	Inflammatory		[52]
Reyes et al.	TNBS	*Il22*^−/−^	Inflammatory		[53]
Eken et al.	CD40 agonistic antibody	anti-IL-22 Ab	Inflammatory	Inflammatory	[54]
Pearson et al.	CD40 agonistic antibody	anti-IL-22 Ab	Not significant		[55]
Pavlidis et al.	*Tbx21*^−/−^ *Rag2*^−/−^ (TRUC)	*Il22*^−/−^	Inflammatory		[56]

**Table 2:** The roles of IL-17 in colitis models

Article	Colitis model	Method	The role of IL-17	The role of IL-23	Ref
Ogawa et al.	DSS	anti-IL-17A Ab	Protective		[63]
Lee et al.	DSS	*Il17a*^−/−^	Protective	Inflammatory	[64]
Yang et al.	DSS	*Il17a*^−/−^	Protective		[65]
	DSS	*Il17f*^−/−^	Inflammatory		
Ito et al.	DSS	*Il17a*^−/−^	Inflammatory		[72]
Maxwell et al.	*Abcb1*^−/− ^+ H.billis	anti-IL-17A Ab	Protective	Inflammatory	[66]
	*Abcb1*^−/−^ + H.billis	anti-IL-17F Ab	Not significant	Inflammatory	
	*Abcb1*^−/−^ + H.billis	anti-IL-17RA Ab	Protective	Inflammatory	
Tachibana et al.	*Il10*^−/−^	*Il17a*^−/−^	Protective		[67]
Morrison et al.	Hh + anti-IL-10	anti-IL-17A Ab	Protective (only in cecum)		[51]
O’Connor et al.	naïve T transfer	*Il17a*^−/−^ T cells	Protective		[68]
Schmidt et al.	naïve T transfer	anti-IL-17A Ab	Inflammatory		[69]
		anti-IL-17F Ab	Not significant		
		anti-IL-17A/F Ab	Inflammatory		
Leppkes et al.	naïve T trasfer	*Il17a*^−/−^ T cells	Not significant		[70]
		*Il17f*^−/−^ T cells	Not significant		
		*Il17f*^−/−^ T cells + anti-IL-17A Ab	Inflammatory		
Zhang et al.	TNBS	*Il17ra*^−/−^	Inflammatory		[71]
Eken et al.	CD40 agonistic antibody	anti-IL-17RA Ab	Not significant	Inflammatory	[54]
Pearson et al.	CD40 agonistic antibody	anti-IL-17A Ab	Not significant		[55]

**Table 3:** The roles of GM-CSF in colitis models

Article	Colitis model	Method	The role of GM-CSF	The role of IL-23	Ref
Pearson et al.	CD40 agonistic antibody	Anti-GM-CSF Ab	Inflammatory	Inflammatory	[55]
Griseri et al.	Naïve T transfer	Anti-GM-CSF Ab	Inflammatory	Inflammatory	[76]
Griseri et al.	Hh + anti-IL-10R	*Csf2rb*^−/−^ or anti-GM-CSF Ab	Inflammatory	Inflammatory	[77]
Han et al.	NSAID-induced ileitis	*Csf2*^*−/−*^*, Csf2rb*^*−/−*^or Anti-GM-CSF Ab	Protective		[81]
Sainathan et al.	DSS	Inject recombinant mouse GM-CSF	Protective		[78]
Xu et al.	DSS	*Csf2*^−/−^	Protective		[79]
Egea et al.	DSS	*Csf2*^−/−^	Protective		[80]

##### IL-22 in colitis

In infection models, the IL-23‒IL-22 axis has been shown to exert protective effects on various enteric pathogens. However, the role of IL-22 in the context of IBD pathology remains controversial (Table 1) [45–56]. Like the phenotype observed in infectious diseases, multiple studies have reported a protective role of IL-22 in colitis induced by dextran sodium sulfate (DSS) treatment or adoptive transfer of naïve CD4^+^ T cells [45–48]. IL-22 is involved in the suppression of colitis through three key biological mechanisms: fortification of epithelial barrier integrity, extracellular matrix secretion, and stimulation of proliferation in gut epithelial stem cells (reviewed by Keir et al. and Arshad et al.) [3, 4]. Furthermore, IL-22 promotes the DNA damage response in the intestinal epithelium and suppresses the development of colorectal cancer, which is often caused by chronic intestinal inflammation in IBD. The targeted depletion of IL-22RA1 from IECs leads to the suppression of apoptosis triggered by DNA damage, consequently leading to increased tumor formation in an inflammation-driven tumor model [57].

Conversely, several reports indicate that IL-22 can exacerbate colitis [49–54, 56]. This phenotype is thought to be related to the activation of a pathway in which IL-22 induces the production of chemokines, such as CXCL1, from IECs, promoting the recruitment of neutrophils to the site of inflammation [50, 54, 56]. Indeed, the depletion of neutrophils improves spontaneous colitis in CX3CR1^Cre^ *Il10ra*^flox/flox^ mice [50]. Additionally, IL-22 has the potential to induce an excessive antimicrobial defense system, including the production of antimicrobial peptides such as RegIII, thereby reducing the diversity of intestinal bacteria [49]. Such dysbiosis could contribute to colitis caused by pathogenic *Helicobacter* spp. While *Helicobacter* spp. remain latent under immunocompetent conditions, *Helicobacter* spp. infection triggers spontaneous colitis in mice with suppressed IL-10 signaling or in immunodeficient mice [52, 58]. In addition, an exaggerated wound healing function mediated by IL-22 can prove detrimental, culminating in tissue fibrosis. Notably, treatment with an anti-IL-22 antibody has been demonstrated to suppress myofibroblast proliferation and subsequent fibrotic symptoms, a common complication of CD, in a trinitrobenzene sulfonic acid (TNBS)-induced colitis model [59].

Given that the elevated IL-22 expression observed in human IBD may play a protective role in mitigating IBD pathology [60, 61], a clinical trial is currently underway in moderate-to-severe UC and CD patients in which an IL-22-Fc fusion protein (UTTR1147A), which acts as an agonist for IL-22R (NCT03558152), is used. The results from Phase 1b of the trial in UC patients demonstrated therapeutic benefits, with a reduction in disease severity compared with that of the placebo groups [62]. Future clinical trials targeting IL-22 signaling pathways in UC and CD patients are expected to uncover key tissue-protective gene expression patterns while also identifying any potential detrimental effects associated with dysbiosis or gut fibrosis.

##### IL-17A/F in colitis

The role of IL-17A/F has been extensively investigated across various colitis models, yet the findings have not provided a definitive consensus (Table 2) [51, 54, 55, 63–72]. Inflammatory responses in models such as the DSS-induced colitis model and *Il10*^−/−^ mice are exacerbated upon inhibition or deficiency of IL-17A [51, 63–68], suggesting a pivotal role for IL-17A in maintaining the integrity of the gut barrier. This effect is attributed to the reinforcement of tight junctions between IECs by IL-17A [64]. Interestingly, while IL-17A inhibition does not affect the expression levels of molecules related to tight junctions, it appears to affect the localization of occludin, a protein that typically colocalizes with F-actin at the apical cell surface. Treatment with an IL-17A antibody leads to the diffusion of occludin, compromising intestinal integrity, increasing gut permeability, facilitating pathogen invasion, and ultimately exacerbating colitis through the altered localization of occludin.

Conversely, certain findings indicate that IL-17A/F can exacerbate colitis induced by adoptive transfer of naïve T cells or TNBS treatment [69–71]. Indeed, blockade of both IL-17A and IL-17F ameliorates colitis, while inhibition of only IL-17A is insufficient. Like IL-22, IL-17A/F is thought to provoke inflammatory responses by acting on IECs to recruit neutrophils and inflammatory monocytes [71, 72]. Interestingly, while blocking IL-17A can exacerbate colitis, perturbation of IL-17F has been reported to reduce disease activity in a DSS-induced colitis model [65]. This dichotomy, despite both IL-17A and IL-17F acting on the same receptor, implies that each may play distinct roles in the initiation and progression of colitis.

Consequently, the pathogenic potential of IL-17A/F in exacerbating colitis has prompted phase 2 studies employing anti-IL-17A or anti-IL-17RA antibodies in the treatment of CD. However, in some patients, this approach results in the exacerbation of colitis [73–75]. This outcome aligns with findings from various mouse models, suggesting that in the context of CD, IL-17A/F may play a protective role in enhancing intestinal barrier integrity.

##### GM-CSF in colitis

The impact of GM-CSF, another cytokine downstream of IL-23, on colitis development has been controversial (Table 3) [55, 76–81]. In experiments involving the cotransfer of CD45.1 WT and CD45.2 *Il23r*^−/−^ naïve T cells into *Rag1*^−/−^ mice, the expression of GM-CSF was significantly reduced in CD45.2 *Il23r*^−/−^ T cells, indicating that IL-23 directly induces GM-CSF production by IL-23R^+^ T cells [76]. Other studies have revealed that antibody blockade or genetic deletion of GM-CSF ameliorates colitis induced by the transfer of naïve T cells, infection with *Helicobacter hepaticas*, and administration of anti-CD40 agonistic antibodies [55, 76, 77], suggesting a highly pathogenic role of GM-CSF in targeting the differentiation of granulocyte‒myeloid progenitors (GMPs) and their effector functions [76]. Notably, GM-CSF strongly promotes the differentiation of eosinophils from GMPs [77], along with their production of IL-6, TNF, and peroxidase, thereby exacerbating colitis. Conversely, the administration of GM-CSF has been demonstrated to suppress inflammatory cytokines such as IL-1 and TNFα by upregulating type I interferon, consequently ameliorating the severity of DSS-induced colitis [78]. Consistent with these findings, GM-CSF^−/−^ mice exhibit exacerbated DSS-induced colitis and nonsteroidal anti-inflammatory drug (NSAID)-induced ileitis [79–81]. Interestingly, these studies suggest that GM-CSF exerts its protective effects directly and autocrinally on IECs, rather than on bone marrow-derived myeloid cells, against DSS-induced colitis or NSAID-induced ileitis by promoting the proliferation of intestinal stem cells and enhancing antiapoptotic pathways, thereby conferring resistance to gut injury [80, 81]. The dual role of GM-CSF, either pathogenic or protective, may be explained by the specific cell types it targets. For example, GM-CSF may exhibit pathogenic effects when it acts on myeloid cells, such as eosinophils, but may have a protective and suppressive role when it targets IECs.

The role of GM-CSF in human IBD is suggested to be protective in CD on the basis of multiple studies examining anti-GM-CSF autoantibodies. Elevated levels of neutralizing anti-GM-CSF autoantibodies have been detected in the serum of CD patients at a significantly greater frequency than in that of UC patients or healthy individuals [82, 83]. Unlike autoantibodies associated with pulmonary alveolar proteinosis, which completely inhibit GM-CSF activity, autoantibodies in CD primarily inhibit glycosylated GM-CSF, resulting in a partial inhibitory effect [83]. Interestingly, CD patients with anti-GM-CSF autoantibodies exhibit dysregulation of the survival and proliferation of IECs, increased intestinal permeability, and reduced bacterial killing activity of neutrophils [81, 84, 85]. Additionally, serum levels of anti-GM-CSF autoantibodies in CD patients are significantly associated with disease activity, such as relapse, ileal/ileocolonic involvement, and an increased risk of complications [82, 83, 86].

By integrating previous findings regarding the Th17-producing cytokines IL-22, IL-17A/F, and GM-CSF, which have been shown to either ameliorate or exacerbate colitis phenotypes (Tables 1–3), we summarize their dual effects: a protective role via direct effects on IECs and a pathogenic role mediated indirectly through myeloid cells (Fig. 4). For example, in a DSS-induced colitis model, IL-22, IL-17A, and GM-CSF play suppressive roles in colitis (Tables 1–3), suggesting their beneficial effects on IECs. Consequently, we propose that the balance or relative strength of the direct versus indirect effects of Th17-producing cytokines may determine the direction of the disease course of IBD patients and their animal models.

Given the substantial heterogeneity in UC and CD regarding disease severity, complications, and treatment resistance, the therapeutic effects of Th17-producing cytokines through supplementation appear to be limited. For example, injections of recombinant proteins such as IL-22-Fc or GM-CSF have demonstrated efficacy in only a subset of IBD patients. Conversely, the adverse effects of IL-17A or IL-17RA inhibitors may also be restricted to a specific subset of patients [62, 73–75, 87–89]. Therefore, it is necessary to elucidate the signaling pathways associated with type 17 immunity that specifically mediate the beneficial effects on IECs in IBD patients. For therapeutic intervention, a combination approach could be employed that enhances the direct actions of Th17-producing cytokines on IECs while simultaneously suppressing their indirect effects on myeloid subsets. This strategy may also involve targeted inhibition of the differentiation and activation of IL-23-producing macrophages.

#### The role of IL-23 in the regulation of CD4^+^ T cells in IBD

The subsequent sections explore the mechanisms by which IL-23 influences the regulation of both pathogenic and regulatory CD4^+^ T cells in the context of IBD.

##### IFN-γ-producing exTh17 cells in colitis

In addition to the signature cytokines of the Th17 lineage, IL-23 has the capacity to induce IFN-γ production by Th17 cells. Compared with other effector Th subsets, Th17 cells are relatively unstable and can adopt traits akin to those of other effector subsets, such as Th1 and Tfh cells [90, 91]. Employing *Il17a*^Cre^ *Rosa26*^YFP^ fate reporter mice [90], we previously demonstrated that IL-17A-producing Th cells can produce IFN-γ alongside IL-17A, transitioning into IFN-γ-producing exTh17 cells that lose IL-17A expression in experimental autoimmune encephalomyelitis (EAE). Notably, IL-23 is indispensable for this conversion of IL-17A^+^ Th17 cells into IFN-γ^+^ exTh17 cells. In a model of Hh-induced colitis following the transfer of naïve T cells, IL-23 plays a pivotal role in the induction of IL-17A^+^ IFN-γ^+^ Th cells rather than IL-17A^+^ IFN-γ^-^ T cells [92–94]. The induction of these IL-17A^+^ IFN-γ^+^ Th cells relies on the transcription factor Rorγt but not Tbet, indicating a deviation of T cells from the Th17 lineage [94].

While the role of IL-12, which is essential for Th1 differentiation, has a limited influence on the manifestation of colitis [95, 96], IFN-γ exacerbates colitis [97, 98]. Furthermore, deficiency of p40 but not p35 influences the expression of IFN-γ [95, 96], indicating that IFN-γ induced by IL-23, rather than IL-12, plays a distinct role in exacerbating colitis. IL-23-driven IL-17A^+^ IFN-γ^+^ Th cells or exTh17 cells are believed to be highly pathogenic T-cell populations implicated in the development and exacerbation of colitis. Further studies are necessary to elucidate a key pathogenic factor downstream of IL-23 signaling expressed by IL-17A^+^ IFN-γ^+^ Th17 cells.

##### IFN-γ^+^ Th1-like cells are distinct from Th17 origin in colitis

Another facet of IL-23R^+^ IFN-γ^+^ Th1-like cells has been elucidated concerning differentiation factors requiring IL-12 and IL-21, which are distinct from Th17 differentiation factors [99]. Upon transfer into *Rag1*^−/−^ mice, in vitro-induced Th1-like cells accumulate in the colon and mediate spontaneous colitis; however, the transfer of *Il23r*^−/−^ Th1-like cells did not result in colitis. These cells, which lack the IL-17A fate reporter marker, are not characteristic of Th17 cells, indicating derivation from a Th1 lineage distinct from neither Th17 nor exTh17 cells. These findings indicate that IL-23 has the potential to act not only on Th17 cells but also on Th1-like cells, conferring a pathogenic function. Furthermore, while the surface marker CD160 is preferentially induced by IL-23 on Th1-like cells, the absence of CD160 abolishes the pathogenic role of Th1-like cells in exacerbating colitis. Mechanistically, compared with WT cells, CD160-deficient Th1-like T cells exhibit increased expression of anti-inflammatory genes such as *Acod1* and *Il10ra*. Taken together, these findings indicate that CD160 induced by IL-23 regulates the expression of anti-inflammatory genes in Th1-like cells, thereby balancing effector functions in inflamed tissues.

##### Inhibitory role of IL-23 in pTreg induction and stability

IL-23 contributes to the pathology of colitis not only by stimulating effector T cells but also by inhibiting the induction or maintenance of peripheral Tregs (pTregs). While deficiency in IL-23 signaling promotes the induction of pTregs derived from naïve T cells in the colon [93], it has been demonstrated that IL-23 acts in a cell-intrinsic manner to directly suppress the induction of pTregs, as shown in a cotransfer experiment with CD45.1 WT and CD45.2 *Il23r*^−/−^ naïve T cells [92]. Furthermore, *Il23a*^−/−^ *Rag2*^−/−^ mice that received *Foxp3*^−/−^ naïve T cells, which are unable to differentiate into pTregs, developed severe colitis, whereas those that received WT naïve T cells did not [93]. Thus, the suppression of pTreg induction is a key mechanism through which IL-23 disrupts gut immune tolerance, thereby promoting the effector functions of pathogenic T cells.

Indeed, Tregs in the gut have been shown to highly express *Il23r* transcripts compared with Tregs in other tissues [100]. IL-23 signaling renders Tregs unstable, decreasing *Abca1* expression, which in turn inhibits liver X receptor signaling within Tregs, ultimately inducing apoptosis. Notably, while Tregs accumulate in the inflamed mucosal tissues of IBD patients, a portion of Tregs actively undergo apoptosis, presumably via a similar mechanism in humans [101, 102].

Considering that Tregs suppress IL-23 production from macrophages [103], IL-23 and Tregs appear to have a mutual inhibitory relationship. Therefore, treatment with anti-IL-23 antibodies, along with increasing the number and function of Tregs, may offer therapeutic potential for IBD patients resistant to IL-23 inhibitors alone and for inducing long-term remission by restoring gut immune tolerance.

#### The cellular origin and regulation of IL-23 in IBD

In a homeostatic state, the expression of IL-23 is tightly regulated by several factors, with IL-10 being a prominent suppressor of IL-23. The absence of IL-10 signaling results in elevated IL-23 expression, thereby activating IL-23R^+^ immune cells that attack the gut’s commensal bacteria or host, ultimately leading to the development of IBD. Notably, *Il10*^−/−^ and *Il10ra*^−/−^ mice spontaneously develop colitis [104, 105]. Additionally, the induction of colitis can be achieved in wild-type mice through the administration of *Helicobacter hepaticus* (Hh) along with anti-IL-10 receptor antibodies, culminating in the onset of colitis within 2–3 weeks [106]. In this model, IL-10 signaling in CX3CR1^+^ macrophages is crucial for preventing spontaneous colitis [107]. Furthermore, deficiency of the IL-10 receptor in CX3CR1^+^ macrophages leads to increased IL-23 expression in these cells [50]. The use of *Il23a* conditional knockout mice that specifically target macrophages further underscores the significant contribution of macrophages as a cellular source of IL-23, exacerbating the onset and progression of colitis in this context. Similarly, CD11c^+^ CX3CR1^+^ macrophages have also been identified as crucial IL-23-producing cells in Hh-induced colitis [108].

CD40 signaling represents another direct stimulatory pathway for IL-23 induction. CD40, a member of the TNF receptor superfamily of membrane proteins, is expressed predominantly by MNPs and B cells. Its activation occurs upon binding to CD154 (CD40L), which is expressed primarily by T cells [109]. Notably, the administration of agonistic antibodies targeting CD40 results in acute colitis in Rag2-deficient mice in an IL-23- and ILC3-dependent manner, accompanied by the accumulation of monocytes and monocyte-derived macrophages, as well as a reduction in CD103^+^ cDC numbers in colonic crypts [110, 111]. Conversely, the depletion of CX3CR1^+^ macrophages with an anti-CSF1R antibody significantly decreases the levels of IL-23 and attenuates the severity of colitis. In this model, CX3CR1^+^ macrophages serve as a cellular source of IL-23, akin to the scenario observed in the IL-10-deficient colitis model [103].

Foxp3^+^ regulatory T cells (Tregs) play a vital role in suppressing IL-23 expression and the development of colitis. It was previously reported that the transfer of naïve T cells without Tregs into *Rag1*^−/−^ or *Rag2*^−/−^ mice causes spontaneous colitis in an IL-23-dependent manner, whereas cotransfer with Tregs prevents the development of colitis [112]. Mechanistically, IL-10 expression by Tregs has been identified as crucial for the prevention of colitis by studies employing *Foxp3*^Cre^ *Il10*^flox/flox^ mice [113, 114]. Since IL-10 has the potential to downregulate CD40 expression in CX3CR1^+^ macrophages [107], Treg-derived IL-10 could mitigate CD40 expression in these cells, thereby suppressing IL-23 production. Additionally, Tregs can repress CD40 signals in macrophages independently of IL-10, leading to decreased IL-23 expression [103]. Adoptive transfer experiments revealed that both WT and *Il10*^−/−^ Tregs significantly suppressed anti-CD40 agonistic antibody-induced colitis. Coculture experiments with CX3CR1^+^ macrophages and Tregs revealed that Tregs downregulate CD40 expression and downstream NF-kB signaling, which are crucial for IL-23 induction. Moreover, the direct interaction between Tregs and CX3CR1^+^ macrophages, which is mediated by the binding of Lag3 to MHCII, results in IL-23 suppression. Thus, multiple mechanisms appear to underlie IL-23 inhibition by Tregs.

While CX3CR1^+^ macrophages have been highlighted as a source of IL-23 in animal models of colitis, the involvement of IL-23-producing cDCs located in tertiary lymphoid tissues, which are crucial during *C. rodentium* infection, in exacerbating IBD pathology remains unclear. Notably, studies have demonstrated that Notch2- or lymphotoxin beta-dependent cDCs play a protective role in dextran sulfate sodium (DSS)-induced colitis [115, 116]. Furthermore, IL-23 has been shown to play a protective role in DSS-induced colitis, which seems contradictory to its pathogenic role observed in human IBD and other mouse models. The seemingly conflicting roles of IL-23 in colitis models could be attributed, in part, to the enhancement of barrier function and wound healing through the IL-23‒IL-22 pathway [115]. However, a previous report indicated that IL-23 exacerbates DSS-induced colitis [117], suggesting that the effects of IL-23 may vary depending on specific environmental conditions and the composition of the gut microbiota. IL-23 production by cDCs may be protective in acute colitis models such as DSS-induced colitis, whereas IL-23 upregulation by CX3CR1^+^ macrophages exacerbates chronic colitis models, including those induced by IL-10 deficiency or anti-CD40 agonistic antibody injection. Further research is needed to elucidate the precise mechanisms underlying the cell type-specific roles of IL-23 in colitis and IBD pathogenesis.

##### Spatiotemporal regulation of IL-23 production by MNPs in the gut

Tissue damage resulting from both acute gut infection by *C. rodentium* and chronic inflammation associated with IBD is highly dependent on IL-23. Although localized and regulated IL-23 production is essential for maintaining intestinal homeostasis and providing protection against infections, dysregulation of IL-23 expression throughout the intestine contributes to IBD pathogenesis. In this context, we discuss the spatiotemporal regulation of IL-23 in models of both acute and chronic gut inflammation.

**Fig. 5:**
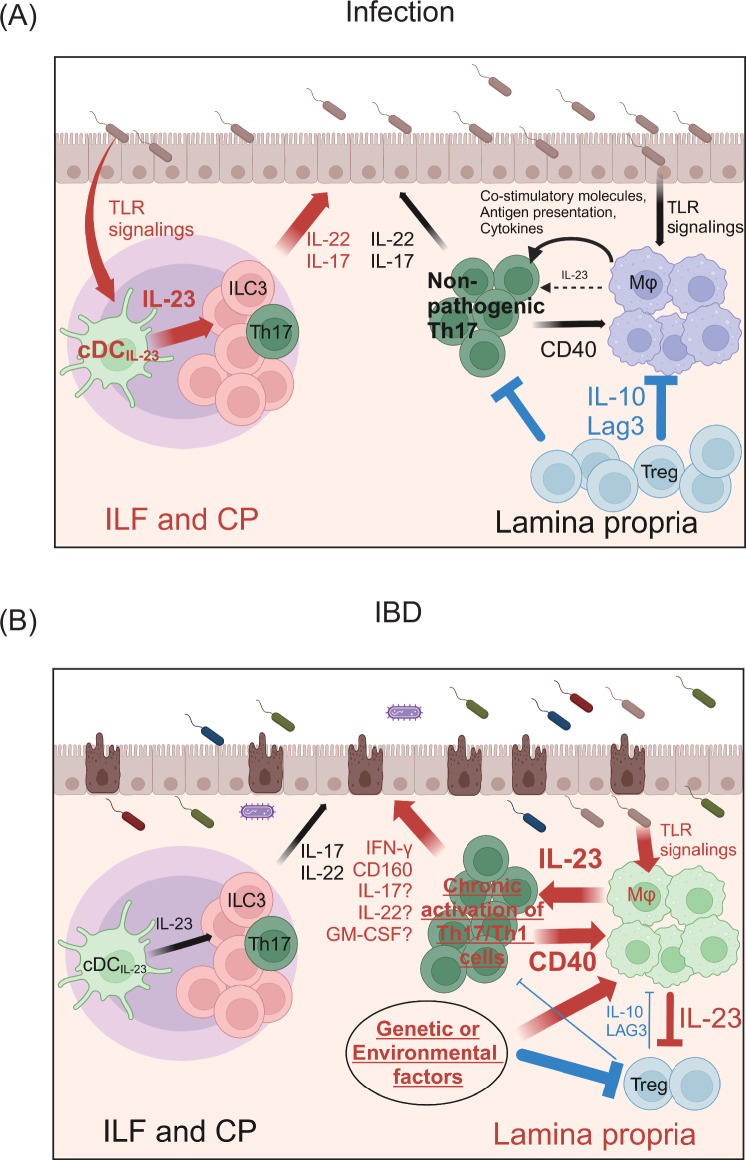
Spatiotemporal regulation of IL-23 expression in mononuclear phagocytes during acute and chronic gut inflammation. **A** In acute inflammation during infection, IL-23 expression is limited to cDC_IL-23_, which is located within GALTs, such as ILFs and CPs. Although macrophages in the lamina propria may also become activated during infection, their production of IL-23 is typically inhibited by IL-10 and Lag3 from Tregs, as well as potentially other environmental factors. Consequently, after elimination of mucosal pathogens, the immune response mediated by type 17 immune cells swiftly returns to baseline levels. **B** In IBD, genetic and environmental factors alter the functionality of macrophages in the lamina propria, either directly or through pathways involving IL-10 and Lag3 from Tregs. These alterations lead to excessive activation of macrophages, which become hypersensitive to CD40 and TLR stimulation from activated T cells and the gut microbiota, respectively. As a result, these macrophages produce large amounts of IL-23. Elevated IL-23 then promotes the differentiation of pathogenic Th17 cells, which drive intestinal inflammation and compromise the barrier function of IECs. IL-23 also impairs the suppressive functions of Tregs, reducing their ability to regulate macrophage activity. This cascade creates a positive feedback loop between T cells and macrophages through the CD40‒IL-23 axis. Continuous stimulation from the gut microbiota, due to impaired barrier function, further augments chronic inflammation driven by type 17 immune responses

The primary distinction in the source of IL-23 production between these two pathologies lies in the specific cell subsets and locations. Under steady-state conditions and during *C. rodentium* infection, IL-23 production is limited to a minor subset of cDCs, defined as EpCAM^+^ DCIR2^+^ CD103^-^ CD11b^-^, termed cDC_IL-23_. These cDC_IL-23_ are predominantly localized within GALTs, such as ILFs and CPs. Thus, IL-23 expression and the subsequent activation of downstream type 17 immune responses, which are mediated by ILC3s and Th17 cells, are compartmentalized within GALTs in response to acute gut infection [8] (Fig. 5A).

In contrast, in IBD, IL-23 production is not confined to cDC_IL-23_ within GALTs. Macrophages located in the lamina propria also produce large amounts of IL-23, leading to widespread effects on IL-23 throughout inflamed gut tissues [50, 108]. This diffuse IL-23 expression, along with the subsequent excessive activation of pathogenic Th17 cells, is considered to drive the pathogenesis of IBD. In this context, IL-23 expression by macrophages is positively regulated by CD40 and TLR signaling. However, in the *C. rodentium* infection model, despite macrophage infiltration into the lamina propria during the late phase of infection and their likely exposure to CD40 and TLR signaling, these macrophages did not produce IL-23. Furthermore, excessive TLR5 signaling induces IL-23 production exclusively in cDC_IL-23_ but not in lamina propria macrophages at steady state [8].

Under physiological conditions, IL-23 expression in macrophages is negatively regulated by inhibitory molecules such as IL-10 and Lag3, which are expressed primarily by Treg cells. However, genetic predispositions and alterations in the gut environment can disrupt this immunological tolerance. When combined with positive signals, such as CD40 and TLR ligation triggered by intestinal infections or dysbiosis, macrophages may gain the capacity to produce excessive IL-23, contributing to IBD pathogenesis (Fig. 5B). Notably, during *C. rodentium* infection, the inhibition of IL-10 production results in severe colitis driven by macrophage-derived IL-23 [118]. A similar phenomenon is observed in IBD models induced by blocking IL-10R and infecting with *Helicobacter hepaticus* [107]. In addition, genetic mutations affecting IL-10 signaling and Treg function in humans have been linked to Mendelian disorder-associated IBD, which is associated with IBD-like intestinal inflammation [43, 119–121]. In patients with IBD, compared with healthy donors, CD14^+^ lamina propria macrophages exhibit significantly greater IL-23 production in response to stimulation with commensal bacteria or TLR agonists [122].

In summary, IL-23 plays both protective and detrimental roles in the gut. IL-23 produced by cDCs in GALTs plays a critical role in maintaining the gut microbiota and defending against infections. In contrast, IL-23 produced by macrophages in the lamina propria contributes to IBD pathogenesis. Although anti-IL-23p19 and p40 antibodies have proven highly effective in treating IBD, more than half of patients exhibit resistance to these therapies [123]. Dysbiosis, often resulting from epithelial barrier disruption and an excessive immune response to commensal bacteria, is believed to drive IBD pathogenesis. However, anti-IL-23 antibody treatment may unintentionally hinder the beneficial role of IL-23 in preserving gut microbiota diversity [115, 116]. Thus, a deeper understanding of the environmental and genetic factors that trigger excessive IL-23 production by macrophages is crucial for developing targeted therapies. Such treatment would aim to selectively inhibit IL-23 production by macrophages, thereby enhancing the efficacy of therapies directed at IL-23 or type 17 immunity.

### Type 17 immunity connects multiple organs in autoimmune diseases

The gut microenvironment is strongly associated with inflammation in extragut tissues, represented by the terms “gut‒joint axis” and “gut‒brain axis”, which are implicated in the pathogenesis of autoimmune conditions such as rheumatoid arthritis (RA) and multiple sclerosis (MS), respectively. These connections have been reviewed extensively elsewhere [124–126]. Given the pathological links between gut immunity and organ-specific inflammation, one possible explanation involves the cross-reactivity of autoimmune T cells with both self-antigens and bacterial peptides.

For example, in RA, two specific autoantigens, N-acetylglucosamine-6-sulfatase (GNS) and filamin A (FLNA), are highly expressed in synovial tissues. Peptides derived from these antigens in the context of HLA-DR have been shown to share significant sequence homology with peptides from intestinal bacteria, potentially originating from *Prevotella* spp., *Parabacteroides* spp., or *Butyricimonas* spp. Furthermore, T cells from RA patients demonstrate a positive correlation between their reactivity to these autoantigens and microbial peptides [127].

Similarly, in MS patients carrying the HLA-DRB3* allele, CD4^+^ T-cell clones expanded from affected brain lesions have shown the ability to respond to self-antigens, such as guanosine diphosphate (GDP)-L-fucose synthase and myelin basic protein, with cross-reactivity to homologous peptides from the gut microbiota [128].

In the following sections, we focus on type 17 immunity, linking gut inflammation to joint and brain inflammation in human diseases and relevant animal models.

#### Link between gut-primed type 17 immune cells and ankylosing spondylitis

Growing evidence indicates that type 17 immune cells primed in the gut play crucial roles in the development of extragut inflammatory disorders, both in humans and various disease models. Ankylosing spondylitis (AS), the most common form of spondyloarthritis (SpA), is a well-studied rheumatic disease in which type 17 immunity interconnects the gut and joint and is believed to be deeply involved in its pathophysiology [129]. AS is characterized by chronic inflammation affecting axial and/or peripheral joints, tendons and entheses, leading to new bone formation [130]. The association of type 17 immune cells with AS pathogenesis is supported by evidence demonstrating the high efficacy of anti-IL-17A biologics in treating AS joint inflammation, as well as the accumulation of ILC3s and mucosal-associated invariant T, γδT and Th17 cells in affected joints [131–134].

In addition to joint inflammation, ~5–10% of AS patients are diagnosed with IBD as an extra-articular manifestation, and notably, up to 50–60% of AS patients are reported to have subclinical intestinal inflammation [135–140]. Although HLA-B27, a human leukocyte antigen class I gene, is currently the strongest genetic risk factor for AS (but not a known risk factor for IBD), several disease-susceptible genes related to type 17 immunity are shared between AS and IBD, such as *IL23R*, *JAK2* and *GPR65* [129, 141, 142]. These observations suggest that AS and IBD share at least some mechanisms underlying their pathogenesis.

Notably, expanded ILC3s in the synovial fluid and blood of AS patients express the key gut homing marker α4β7, which is commonly expressed by ILC3s residing in the gut, suggesting that gut ILC3s may migrate into the joint tissue through the bloodstream [131]. However, joint ILC3s in SpA patients have been reported to predominantly produce IL-22 and/or GM-CSF rather than IL-17, the signature cytokines of gut ILC3s, indicating functional alterations between ILC3s in the gut and joint tissues [133, 143].

With respect to T-cell antigen specificity in the gut-joint axis, a subset of CD8^+^ T cells expressing TCRs paired with TRAV21 and TRBV9 and carrying an AS-associated TRBV9-CDR3 motif were clonally expanded in the synovial fluid of HLA-B27^+^ AS patients. Furthermore, CD8^+^ SKW-3 cells transduced with these specific TCR components became activated in vitro not only in response to self-peptides but also in response to bacterial peptides presented in the context of HLA-B27 expressed by K562 cells.

The TCR specificity for gut and joint antigens in an HLA-B27-dependent manner provides crucial insights into understanding multiorgan inflammatory disorders that concurrently manifest as AS and IBD. It is conceivable that potentially arthritogenic T cells residing in the gut may be primed upon encountering certain bacteria along with inflammatory cues, leading to extragut inflammation in response to joint self-antigens [144]. Although it remains uncertain whether CD8^+^ T cells with these TCRs serve as a pathogenic source of IL-17 in the inflamed joints of AS patients, this scenario is possible since the number of IL-17-producing CD8^+^ T (Tc17) cells is greater in the gut of IBD patients than in that of healthy controls [145–147].

Furthermore, selective depletion of TRBV9^+^ T cells via the administration of a cytotoxic humanized anti-TRBV9 monoclonal antibody (BCD180) demonstrated dramatic efficacy in achieving and maintaining disease remission in an anti-TNF therapy-resistant HLA-B27^+^ AS patient with blood T cells harboring the AS-associated TRBV9-CDR3 motif. This single case report suggests a crucial role of the antigen specificity of TRBV9^+^ T cells in the development and maintenance of HLA-B27^+^ AS. Since BCD180 has been proven tolerable and safe without any major adverse events, a phase II trial using BCD180 is currently underway (NCT05445076) [148].

On the basis of these findings, it has been proposed that type 17 immune responses to certain gut bacteria may occur before joint inflammation in AS. However, given that a certain proportion of AS patients lack gut inflammation and are resistant to treatment with IL-23 blockade, which is effective for IBD treatment, the pathogenesis of AS also appears to exhibit substantial heterogeneity [149]. Thus, despite the apparent linkage of the underlying mechanism between the gut and joint inflammation, simultaneous multiorgan inflammation may also operate independently and differentially under distinct tissue-specific mechanisms.

#### Animal models of autoimmune arthritis mediated by gut-primed type 17 immune cells

Several animal models support the link between gut type 17 immunity and joint inflammation. SKG mice, a model of Th17 cell-dependent autoimmune arthritis, carry a hypomorphic point mutation in *Zap70* on a BALB/c genetic background, resulting in the production of arthritogenic T cells in the thymus due to altered thymic selection [150, 151]. Arthritis in SKG mice can be induced by promoting Th17 cell differentiation in the periphery through stimulating innate immunity with fungal components such as zymosan and mannan. Pathogenic Th17 cells are abundantly localized in the joints of arthritic SKG mice, and IL-17A production by Th17 cells serves as the primary factor for arthritis development, as evidenced by studies in *Il17a*^−/−^ SKG mice or adoptive transfer models in which *Il17a*^−/−^ T cells were used [151–153]. Treatment with an anti-IL-23 antibody was also effective in both the prevention and treatment phases of arthritis, indicating the essential role of the IL-23/IL-17A axis in this disease model [154, 155].

Recent studies have revealed a profound association between gut commensal bacteria and arthritis development in SKG mice. Intriguingly, mice bred under germ-free (GF) conditions are resistant to arthritis induction by zymosan injection. *Prevotella copri* (*P. copri*) was found to be a unique bacterium enriched in the feces of RA patients compared with healthy controls. Notably, GF SKG mice, which were transferred with a human RA fecal sample containing *P. copri*, exhibited increased differentiation of Th17 cells in the large intestine, increasing arthritis susceptibility. Moreover, in vitro studies have shown that *P. copri* has a greater capacity to induce IL-6 and IL-23 production from bone marrow DCs than other commensal bacteria, such as *Escherichia coli* or *Bacteroides fragilis*, suggesting that a particular bacterium is sufficient to promote the differentiation of Th17 cells with arthritogenic potential in the gut. Gut-primed Th17 cells subsequently migrate to joint tissues and begin to react with joint self-antigens, where they mediate synovial inflammation [156].

K/BxN and CIA models are commonly used to study autoimmune arthritis, which are characterized by the production of pathogenic autoantibodies against glucose-6-phosphate isomerase (GPI) and type II collagen (CII), respectively. These models exhibit varying degrees of association between gut type 17 immunity and arthritis development [157–163]. Like SKG mice, GF K/BxN mice are resistant to arthritis, accompanied by a significant reduction in serum autoantibody levels. However, upon mono-colonization of GF K/BxN mice with SFB, the ability to produce anti-GPI autoantibodies and trigger arthritis is restored in gnotobiotic K/BxN mice. A proportion of Th17 cells in the spleen of gnotobiotic K/BxN mice express α4β7, indicating that SFB-induced Th17 cells likely shuttle from the gut to the spleen. These factors help promote germinal center (GC) formation and autoantibody production through the direct effect of IL-17 on B cells within the GC [159].

In another report, the IL-23/Th17 axis was demonstrated to play a crucial role in conferring pathogenic functions on autoantibodies in K/BxN and CIA models. Mechanistically, during the onset of arthritis, IL-23 promotes IL-21 and IL-22 production by Th17 cells, which suppresses *St6gal1* expression in plasma cells, resulting in increased levels of the pathogenic form of IgG glycosylation. Conversely, the majority of CII-specific autoantibodies in *Il23a*^−/−^ CIA mice remain highly sialylated, and immunocomplexes (ICs) comprising sialylated autoantibodies appear to have a weaker capacity to induce proinflammatory cytokines such as TNF-α and IL-6 from BMDCs compared to ICs comprising glycosylated autoantibodies from WT CIA mice.

Similarly, the arthritogenic capacity of serum IgG from K/BxN mice treated with IL-23 inhibitors is significantly lower than that of serum IgG from control K/BxN mice. However, the pathogenicity of IgG autoantibodies from K/BxN mice following IL-23 neutralization can be fully restored by removing sialic acid from serum IgG with neuraminidase treatment, suggesting the reduced pathogenicity of sialylated antibodies. The transfer of the serum after treatment into recipient mice effectively causes arthritis, indicating a shared mechanism enhancing the pathogenic functions of autoantibodies via the transformation of sialylated IgG into a glycosylated state via the IL-23/Th17 axis in both arthritis models [160]. Taken together, in arthritis models in which microbiome-induced Th17 cells play a crucial role in disease progression, gut-primed Th17 cells may exhibit direct pathogenicity in joint tissues and indirectly contribute to disease development by promoting GC formation in lymphoid organs to upregulate autoantibody production and modifying autoantibodies from a sialylated state to a glycosylated state with greater proinflammatory potential.

#### Therapeutic perspective in arthritis via the targeting of type 17 immunity

As evidenced by studies demonstrating the crucial role of IL-17A in the pathogenesis of autoimmune arthritis in mice, IL-17A blockade was initially considered a promising treatment strategy for human RA. However, in sharp contrast to the robust efficacy of anti-IL-17A therapy in AS, IL-17A blockade unexpectedly showed limited efficacy in ameliorating RA [164–167]. This enigma may be attributed, at least in part, to the conversion of arthritogenic Th17 cells in RA-affected joints into Th1-like cells that also produce a type 17 cytokine, GM-CSF, soon after the onset of joint inflammation. This implies that the efficacy of IL-17A blockade could be limited to the very early stage of the disease [168–171].

Furthermore, a recent report provided novel insight into the regulation of the inflammatory signaling cascade downstream of IL-17A. Conventionally, IL-17A signaling facilitates the formation of canonical Act1‒TRAF5 complexes to exert its proinflammatory effects; however, chronic IL-17A stimulation increases the expression of Src homology 2 domain-containing tyrosine phosphatase (SHP2), which can directly interact with dephosphorylated Act1, forming noncanonical Act1‒SHP2 complexes. These Act1-SHP2 complexes supplant Act1-TRAF5 complexes, thereby initiating IL-17R signaling autonomously even in the absence of IL-17A stimulation. In contrast to the conventional “front door” pathway, through which classical Act1-TRAF5 complexes transduce IL-17R signaling in an IL-17A-dependent manner, this autonomous activation could perpetuate inflammation, thereby circumventing the effect of IL-17A neutralization. This alternative activation mechanism downstream of IL-17R signaling contributes partially to the ineffectiveness of IL-17A blockade in certain IL-17A-related human disorders. Notably, iguratimod, an antirheumatic drug used in Japan and China, has the potential to inhibit the interaction between Act1 and SHP2, thus ameliorating the progression of CIA by mitigating the production of type 17 cytokines and their downstream chemokines [172].

#### Neuroinflammation mediated by gut-primed type 17 immune cells

Neuroinflammation is characterized by a chronic form of type 17 inflammation affecting the brain and spinal cord, often displaying a notable correlation with type 17 immune cells primed in the gut. MS is a highly heterogeneous autoimmune disorder characterized by demyelinating lesions within the central nervous system (CNS), exhibiting a broad spectrum of clinical disease courses [173]. Although the specific etiology of MS remains elusive, type 17 cytokines, such as IL-17A and IL-22, have been implicated in potentially increasing the permeability of the blood‒brain barrier (BBB). The observation of enriched Th17 cells within active MS brain lesions crossing the BBB, alongside the positive correlation between IL-17A production by myelin basic protein-primed CD4^+^ T cells from peripheral blood mononuclear cells (PBMCs) and disease activity, has implicated type 17 immunity in the pathogenesis of MS [174–176].

EAE, a murine model of MS, has been widely used for investigating MS pathology. Among the various proinflammatory cytokine networks, the IL-23/IL-17A axis plays a crucial role in the pathogenesis of EAE development [177–180]. A notable correlation is observed between the abundance of intestinal Th17 cells and disease activity in MS patients, as similarly demonstrated by the protective effect against EAE in GF mice [181]. Notably, mono-colonization of SFB in GF mice restores the induction of intestinal Th17 cells, leading to susceptibility to EAE development [181, 182].

A recent study revealed the distinct roles of these two gut microorganisms in synergistically promoting the generation of encephalitogenic Th17 cells in the intestine of an EAE model. Specifically, UvrABC system protein A, a constituent of *Lactobacillus reuteri* (*L. reuteri*), has been identified as a molecular mimic of the MOG peptide, thereby triggering mild activation of MOG-specific T cells in the gut. These gut-primed T cells undergo further differentiation into Th17 cells upon exposure to IL-23 and SAA, both of which are produced by gut DCs that sense bacteria of the Erysipelotrichaceae family (OTU0002). Notably, upon cocolonization of *L. reuteri* with OTU0002, EAE is fully induced in gnotobiotic mice, whereas GF mice mono-colonized with *L. reuteri* fail to develop EAE, similar to control GF mice. These findings underscore the requirement for two distinct signals from intestinal bacteria, including potential antigenicity, such as molecular mimicries associated with the CNS, as well as environmental cues that drive Th17 cell differentiation in the gut [183].

Another study demonstrated the role of the gut as a reservoir of pathogenic Th17 cells. Through the combined analysis of scRNA-seq and TCR-seq data from Th17 cells from various tissues under steady-state conditions and during the development of EAE, a subset of stem-like SLAMF6^+^ IL-17A^+^ Th17 cells was identified. These cells traffic to the intestine and are maintained by the microbiota. During the progression of EAE, these cells acquire proinflammatory functions via IL-23 signaling in the spleen and undergo preferential conversion into CXCR6^+^ IFN-γ^+^ GM-CSF^+^ Th17 cells, which subsequently migrate into the CNS. These findings provide novel insight into the role of the gut microbiota in contributing to disease by maintaining a reservoir of stem-like Th17 cells, which then give rise to pathogenic Th17 cells in the spleen rather than directly inducing pathogenic Th17 cells in the gut [184].

An interesting report has also highlighted the role of ILC3s in modulating neuroinflammation as bona fide antigen-presenting cells [185]. The accumulation of ILC3s was observed in the inflamed spinal cords of EAE, where they are recruited and mature along with the upregulation of MHC II expression. Functionally, these ILC3s serve as antigen-presenting cells, facilitating the activation of encephalitogenic T cells [185]. The specific origin of the expanded ILC3s within the CNS remains unclear; however, it is reasonable to speculate that some of these ILC3s may originate from the gut, on the basis of evidence indicating that ILC3s are typically enriched in the gut and that those gut-derived ILCs have the capacity to circulate to distant organs, thereby contributing to tissue inflammation [186].

#### Dietary habits enhancing type 17 immunity

Daily dietary habits significantly influence the gut environment, thereby profoundly impacting gut type 17 immunity and its associated diseases. The consumption of a high-salt diet (HSD) has been shown to induce the expression of serum glucocorticoid kinase 1 (SGK1), a serine/threonine kinase, in Th17 cells. SGK1 activation promotes the expression of IL-23R by deactivating Foxo1, a negative regulator of Th17 cells. Consequently, SGK1 activation in Th17 cells stabilizes their phenotype and enhances pathogenic GM-CSF production, thereby exacerbating neuroinflammation in EAE [187, 188].

Furthermore, another study revealed the effect of high salt intake on the gut microbiota and its association with the development of autoimmune diseases. Compared with normal salt diet (NSD)-fed control mice, HSD-fed mice presented an exacerbated disease course of EAE. Mechanistically, a HSD significantly reduced the proportion of *Lactobacillus murinus* (*L. murinus*), a bacterium known to metabolize tryptophan into indoles, including indole-3-lactic acid (ILA), which inhibits Th17 cell differentiation, in the gut. Indeed, the fecal ILA concentration was decreased, whereas the frequency of Th17 cells in the SI-LP and spinal cord was increased in HSD-fed mice compared with those in NSD-fed mice. Furthermore, *L. murinus* supplementation during HSD consumption ameliorated the severity of EAE, suggesting that HSD promoted pathogenic Th17 differentiation in the gut through a reduction in *L. murinus*-derived ILA [189].

## Concluding remarks

The complex interplay among type 17 immune components, including IL-23, IL-17, IL-22, and GM-CSF, underscores their pivotal roles in both mucosal host defense and autoimmune pathogenesis. Despite recent progress, the precise roles and spatiotemporal regulation of these components, especially IL-23, in autoimmune settings remain incompletely understood relative to those of IL-17, IL-22, and GM-CSF. Therefore, unraveling the dynamics of IL-23 production from key MNPs such as cDCs and macrophages and its context-dependent regulation in chronic inflammation versus acute mucosal infections is crucial for a comprehensive understanding of IBD and autoimmunity pathogenesis.

Notably, IL-17 production by gut Th17 cells manifests distinct functions independent of IL-23, which is crucial for maintaining gut immune homeostasis, including microbiota composition. Conversely, the physiological and pathological expression of IL-22 and GM-CSF predominantly relies on IL-23 in various inflammation models, highlighting the necessity of discerning which pathogenic cytokine pathways to target while safeguarding their physiological roles. Investigating specific intracellular signaling events mediated by type 17 cytokines in physiological versus pathological contexts may facilitate the development of targeted therapeutic interventions.

Moreover, considering the role of type 17 immunity in chronic inflammation in multiple organs is essential. While various factors, such as molecular mimicry and environmental cues, contribute to multiorgan inflammation, the necessity of relevant foreign antigens for sustaining autoimmune responses during chronic phases remains uncertain. If the potential perpetuation of Th17 activation occurs through molecular mimicry, it will be important to consider the need to target not only effector cytokines but also relevant foreign antigens to effectively manage chronic inflammation and maintain autoimmune remission.
